# Clinical and Biological Correlates of Emotional Dysregulation in Children and Adolescents: A Transdiagnostic Approach to Developmental Psychopathology

**DOI:** 10.3390/brainsci14080782

**Published:** 2024-07-31

**Authors:** Annarita Milone, Gianluca Sesso

**Affiliations:** 1 Department of Child and Adolescent Psychiatry and Psychopharmacology, IRCCS Stella Maris Foundation, Viale del Tirreno 331, 56128 Pisa, Italy; annarita.milone@fsm.unipi.it; 2 Social and Affective Neuroscience Group, Molecular Mind Lab, IMT School for Advanced Studies, Piazza San Francesco, 55100 Lucca, Italy

## 1. Introduction

Emotion regulation may be defined as the ability to regulate behavioral and physiological reactivity to sensory stimuli and environmental situations. It entails any kind of strategy that aims to monitor, assess, and modulate emotions in the context of various conditions [1]. On the other hand, the failure to regulate one’s own emotions, that is, Emotional Dysregulation (ED), has become a diagnostic challenge in recent decades due to the great heterogeneity of clinical presentations and the various definitions of the construct proposed; it has also been recently considered a core dimension of psychopathology in youths in a trans-nosographic conceptualization.

In childhood and adolescence, ED affects at least 1–6% of the general population, and significantly and negatively impacts school functioning and professional outcomes, social adjustment and acceptability by peers, and current and later quality of life [2]. For these reasons, ED represents a highly relevant construct in psychiatry research and clinical practice in terms of developmental outcomes and prognostic implications [3]. In light of this, clinicians should always aim to detect the presence of ED when dealing with challenging children and adolescents by means of several validated clinical measures (e.g., [4]). In addition, neurofunctional findings based on brain imaging techniques and peripheral indexes regarding the functioning of the autonomic nervous system have recently emerged as reliable transdiagnostic biomarkers of ED in psychopathology [5].

The principal aim of this Special Issue was thus to address some major aspects of ED in youths: (1) the etiology and early precursors of ED in youth; (2) the developmental trajectories of ED from early childhood to adulthood; (3) the clinical presentation, comorbidities and correlates of ED in youth, including irritability and suicidality; (4) the clinical assessment of ED by means of standardized measures; and (5) psychosocial interventions, psychotherapy, and pharmacological treatment options. In this Special Issue, six relevant contributions, comprising four original articles, one systematic review, and one narrative review that provide significant insights into the clinical assessment and management of ED in youth, were included (see Table A1). This Special Issue gathers different perspectives on ED across varying contexts, highlighting its significant implications for mental health.

## 2. Summary of Included Studies

Conti and colleagues [6] conducted a systematic review examining the interplay between ED and post-traumatic stress symptoms in adolescents and young adults. The authors found that ED may represent a risk factor that mediates the vulnerability of young people to the development of stress symptoms after trauma exposure. Although most included studies reported inconclusive findings, this systematic review emphasizes the importance of developing emotion regulation strategies as part of Post-Traumatic Stress Disorder (PTSD) prevention programs.

Brancati and coworkers [7] focused on the developmental trajectory of Attention-Deficit/Hyperactivity Disorder (ADHD), particularly regarding early referral to specialty care or treatment with Methylphenidate. They found that early Methylphenidate treatment is associated with reduced ED symptoms and lower rates of mood disorders in adulthood, with negative emotionality mediating the relationship between pharmacotherapy and comorbidity. They also reported higher rates of past disruptive behaviors and current externalizing features in patients with early referral during childhood. Taken together, these findings highlight the fact that ED, especially its externalizing component, can be regarded as a core target of ADHD treatment.

Serra et al. [8] explored the factors associated with high irritability scores in early-onset mood disorders using the Affective Reactivity Index. They identified severe ED as a key factor influencing both parent and self-rated irritability scores, highlighting its importance in differentiating diagnostic profiles and treatment approaches in adolescent mood disorders.

Cerniglia’s workgroup [9] employed a network analysis approach to study risk-taking and self-harm behaviors in typically developing adolescents. This comprehensive nomological approach linked these behaviors to deficits in emotion regulation strategies and maladaptive psychological functioning. Their findings highlight the critical role of effective emotion regulation strategies to mitigate risk-taking and self-harm behaviors, thus informing psychosocial interventions and prevention strategies targeting adolescents engaging in risky conducts.

Masi and colleagues [10] conducted a longitudinal prospective study on non-suicidal self-injury and suicidality in adolescents with mood disorders, revealing that persistent self-injury is associated with higher levels of cyclothymic temperament and an increased risk of suicidal behavior. The authors also found that, at follow-up, patients with persistent and previous self-harming behaviors were more severely impaired than those with no such symptoms; the internalizing problems and ED of these patients also failed to improve. These findings support a continuity between self-injury and suicidality, suggesting the prognostic validity of suggesting that the persistence of symptoms is associated with higher ED and cyclothymic features. The authors concluded by highlighting the need for targeted interventions that address ED and mood symptoms to prevent escalation from self-injury to suicidal ideation and behavior.

Finally, Easdale-Cheele et al. [11] conducted a comprehensive, updated, narrative review summarizing the available findings related to the multimodal interventions targeting ED across the lifespan. They discussed the neurobiological, neuropsychological, and psychosocial factors that influence ED, highlighting both pharmacological and non-pharmacological treatment approaches aimed at enhancing emotional resilience. They also provided evidence with important implications for clinical practice and future research in this field.

## 3. Conclusions

These six relevant contributions underscore the complex interplay between ED symptoms, psychiatric comorbidities, and developmental trajectories in adolescents and young adults. It is now clear that ED is implicated in the development of post-traumatic stress symptoms, ADHD, irritability in mood disorders, risk-taking behaviors, and non-suicidal self-injury. The authors of these articles propound the need for tailored interventions that address emotion regulation as a pivotal component of therapeutic strategies across various mental health disorders. The findings collectively emphasize the importance of developing targeted interventions to improve ED across various diagnostic profiles, thereby enhancing psychological functioning and mitigating risk behaviors.

## Appendix A

**Table A1:** Summary of included studies.

Ref	Authors	Country	Institution	Type
[6]	Lorenzo Conti, Sara Fantasia, Miriam Violi, Valerio Dell’Oste, Virginia Pedrinelli and Claudia Carmassi	Italy	University of Pisa	Systematic Review
[7]	Giulio Emilio Brancati, Ugo De Rosa, Francesco De Dominicis, Alessandra Petrucci, Alessandro Nannini, Pierpaolo Medda, Elisa Schiavi and Giulio Perugi	Italy	University Hospital of Pisa, University of Pisa, Mental Health Center of Spoleto	Original Article
[8]	Giulia Serra, Massimo Apicella, Elisa Andracchio, Giorgia Della Santa, Caterina Lanza, Monia Trasolini, Maria Elena Iannoni, Gino Maglio and Stefano Vicari	Italy	IRCCS Bambino Gesù Children’s Hospital of Rome, Catholic University of the Sacred Heart of Rome	Original Article
[9]	Luca Cerniglia, Silvia Cimino, Renata Tambelli and Marco Lauriola	Italy	International Telematic University “Uninettuno” of Rome, University of Rome “Sapienza”,	Original Article
[10]	Gabriele Masi, Simone Pisano, Gianluca Sesso, Cristina Mazzullo, Stefano Berloffa, Pamela Fantozzi, Antonio Narzisi, Francesca Placini, Elena Valente, Valentina Viglione and Annarita Milone	Italy	IRCCS Fondazione Stella Maris of Pisa, IMT School for Advanced Studies of Lucca, University of Napoli “Federico II”,	Original Article
[9,11]	Thomas Easdale-Cheele, Valeria Parlatini, Samuele Cortese and Alessio Bellato	UK, USA, Italy and Malaysia	University of Southampton, New York University, University of Bari “Aldo Moro”, University of Nottingham Malaysia	Narrative Review
